# Supplementary material for: Moral Foundational Framing and Its Impact on Attitudes and Behaviours
Source: Behav Sci (Basel). 2022 Apr 20;12(5):118. doi: 10.3390/bs12050118 (PMC9137563; doi:10.3390/bs12050118)
Supplement: Supplementary file 1 [file behavsci-12-00118-s001.zip › behavsci-1435441-supplementary.pdf]

Analysis S1

Binomial Logistic Regression (software used [32–34])

Model Fit Measures

Model	Deviance	AIC	R^2_{McF}
1	.	.	.

Model Coefficients - …

Predictor	Estimate	SE	Z	p
Intercept	.	.	.	.
Condition:				
1 – 0	.	.	.	.
2 – 0	.	.	.	.
3 – 0	.	.	.	.
4 – 0	.	.	.	.
CareMean	.	.	.	.
FairMean	.	.	.	.
LoyalMean	.	.	.	.
AuthMean	.	.	.	.
SancMean	.	.	.	.

Note. Estimates represent the log odds of …

Ordinal Logistic Regression

Model Fit Measures

Model	Deviance	AIC	R^2_{McF}
1	3244	3264	0.0888
2	3236	3264	0.0909
3	3180	3248	0.1066

Note. The dependent variable 'AttitudeTotal' has the following order: 0 | 1 | 2 | 3 | 4 | 5

Model Comparisons

Comparison					
Model		Model	χ^2	df	p
1	-	2	7.36	4	0.118
2	-	3	55.96	20	< .001

Model Specific ResultsModel 1Model 2Model 3

Model Coefficients - AttitudeTotal

		95% Confidence Interval						95% Confidence Interval	
Predictor	Estimate	Lower	Upper	SE	Z	p	Odds ratio	Lower	Upper
SancMean	-0.0209	-0.179	0.1371	0.0805	-0.260	0.795	0.979	0.836	1.147
AuthMean	-0.6399	-0.843	-0.4382	0.1032	-6.202	<.001	0.527	0.430	0.645
LoyalMean	-0.2172	-0.406	-0.0289	0.0962	-2.258	0.024	0.805	0.666	0.971
FairMean	0.8256	0.606	1.0481	0.1128	7.320	<.001	2.283	1.833	2.852
CareMean	0.4363	0.233	0.6394	0.1035	4.214	<.001	1.547	1.263	1.895

[3]

Model Coefficients - AttitudeTotal

		95% Confidence Interval						95% Confidence Interval	
Predictor	Estimate	Lower	Upper	SE	Z	p	Odds ratio	Lower	Upper
SancMean	-0.0211	-0.179	0.1378	0.0809	-0.26035	0.795	0.979	0.836	1.148
AuthMean	-0.6425	-0.847	-0.4398	0.1037	-6.19527	<.001	0.526	0.429	0.644
LoyalMean	-0.2217	-0.411	-0.0331	0.0964	-2.30088	0.021	0.801	0.663	0.967
FairMean	0.8040	0.583	1.0278	0.1135	7.08479	<.001	2.234	1.791	2.795
CareMean	0.4531	0.249	0.6577	0.1043	4.34562	<.001	1.573	1.282	1.930
Condition:									
0 – 4	-0.0147	-0.368	0.3384	0.1802	-0.08178	0.935	0.985	0.692	1.403
1 – 4	-2.57e–4	-0.343	0.3423	0.1747	-0.00147	0.999	1.000	0.710	1.408
2 – 4	-0.3630	-0.703	-0.0233	0.1734	-2.09279	0.036	0.696	0.495	0.977
3 – 4	-0.2543	-0.600	0.0911	0.1763	-1.44235	0.149	0.775	0.549	1.095

[3]

Model Coefficients - AttitudeTotal

		95% Confidence Interval						95% Confidence Interval	
Predictor	Estimate	Lower	Upper	SE	Z	p	Odds ratio	Lower	Upper
SancMean	-0.1418	-0.49803	0.2187	0.183	-0.777	0.437	0.86777	0.6077	1.2445
AuthMean	-0.0671	-0.50133	0.3649	0.221	-0.304	0.761	0.93509	0.6057	1.4403
LoyalMean	-0.5016	-0.86671	-0.1387	0.185	-2.706	0.007	0.60555	0.4203	0.8705
FairMean	0.5052	0.05287	0.9657	0.232	2.176	0.030	1.65725	1.0543	2.6267
CareMean	0.1645	-0.25416	0.5734	0.210	0.782	0.434	1.17877	0.7756	1.7743
Condition:									
0 – 4	-4.8966	-7.28036	-2.5526	1.204	-4.066	<.001	0.00747	6.89e-4	0.0779
1 – 4	-2.0717	-4.23707	0.0639	1.095	-1.891	0.059	0.12598	0.0144	1.0659
2 – 4	-1.9870	-3.90728	-0.0979	0.970	-2.048	0.041	0.13711	0.0201	0.9067
3 – 4	-1.3164	-3.44959	0.8086	1.085	-1.213	0.225	0.26810	0.0318	2.2447
SancMean ┬ Condition:									
SancMean ┬ (0 – 4)	0.1575	-0.37140	0.6855	0.269	0.585	0.559	1.17054	0.6898	1.9847
SancMean ┬ (1 – 4)	0.4079	-0.10472	0.9213	0.262	1.560	0.119	1.50371	0.9006	2.5127
SancMean ┬ (2 – 4)	-0.1055	-0.60068	0.3855	0.251	-0.420	0.675	0.89990	0.5484	1.4703
SancMean ┬ (3 – 4)	0.1180	-0.39243	0.6262	0.260	0.454	0.650	1.12522	0.6754	1.8705
AuthMean ┬ Condition:									
AuthMean ┬ (0 – 4)	-0.9087	-1.57249	-0.2489	0.337	-2.695	0.007	0.40305	0.2075	0.7797
AuthMean ┬ (1 – 4)	-0.9762	-1.62193	-0.3322	0.329	-2.970	0.003	0.37675	0.1975	0.7174
AuthMean ┬ (2 – 4)	-0.3476	-0.94384	0.2486	0.304	-1.144	0.253	0.70637	0.3891	1.2822
AuthMean ┬ (3 – 4)	-0.9449	-1.58613	-0.3068	0.326	-2.899	0.004	0.38873	0.2047	0.7358
LoyalMean ┬ Condition:									
LoyalMean ┬ (0 – 4)	0.6192	0.00707	1.2302	0.312	1.986	0.047	1.85741	1.0071	3.4221
LoyalMean ┬ (1 – 4)	0.1354	-0.41576	0.6844	0.280	0.483	0.629	1.14494	0.6598	1.9825
LoyalMean ┬ (2 – 4)	0.5155	-0.06827	1.1018	0.298	1.729	0.084	1.67448	0.9340	3.0097
LoyalMean ┬ (3 – 4)	0.4045	-0.16984	0.9808	0.293	1.379	0.168	1.49852	0.8438	2.6667
FairMean ┬ Condition:									
FairMean ┬ (0 – 4)	0.7131	-0.00114	1.4254	0.363	1.963	0.050	2.04040	0.9989	4.1595
FairMean ┬ (1 – 4)	0.4633	-0.22088	1.1541	0.350	1.323	0.186	1.58937	0.8018	3.1713
FairMean ┬ (2 – 4)	0.2410	-0.40741	0.8932	0.331	0.728	0.467	1.27256	0.6654	2.4429
FairMean ┬ (3 – 4)	0.3182	-0.38230	1.0188	0.357	0.891	0.373	1.37465	0.6823	2.7698
CareMean ┬ Condition:									
CareMean ┬ (0 – 4)	0.7506	0.11304	1.3994	0.328	2.290	0.022	2.11818	1.1197	4.0528
CareMean ┬ (1 – 4)	0.4756	-0.16094	1.1169	0.325	1.462	0.144	1.60891	0.8513	3.0553

Model Coefficients - AttitudeTotal

Predictor	Estimate	95% Confidence Interval		SE	Z	p	Odds ratio	95% Confidence Interval	
		Lower	Upper					Lower	Upper
CareMean ┬ (2 – 4)	0.1787	-0.43507	0.7898	0.311	0.574	0.566	1.19563	0.6472	2.2031
CareMean ┬ (3 – 4)	0.3126	-0.31734	0.9455	0.321	0.973	0.331	1.36700	0.7281	2.5740

[3]

References

[32] The jamovi project (2020). *jamovi*. (Version 1.2) [Computer Software]. Retrieved from https://www.jamovi.org.

[33] R Core Team (2019). *R: A Language and environment for statistical computing*. (Version 3.6) [Computer software]. Retrieved from https://cran.r-project.org/.

[34] Ripley, B., Venables W., Bates, D. M., Hornik, K., Gebhardt, A., & Firth, D. (2018). *MASS: Support Functions and Datasets for Venables and Ripley's MASS*. [R package]. Retrieved from https://cran.r-project.org/package=MASS.

Analysis S2

Binomial Logistic Regression (software used [32–34])

Model Fit Measures

Model	Deviance	AIC	R^2_{McF}
1	.	.	.

Model Coefficients - …

Predictor	Estimate	SE	Z	p
Intercept	.	.	.	.
Condition:				
1 – 0	.	.	.	.
2 – 0	.	.	.	.
3 – 0	.	.	.	.
CareMean	.	.	.	.
FairMean	.	.	.	.
LoyalMean	.	.	.	.
AuthMean	.	.	.	.
SancMean	.	.	.	.

Note. Estimates represent the log odds of …

Ordinal Logistic Regression

Model Fit Measures

Model	Deviance	AIC	R^2_{McF}
1	2528	2548	0.102
2	2522	2548	0.104
3	2495	2551	0.113

Note. The dependent variable 'AttitudeTotal' has the following order: 0 | 1 | 2 | 3 | 4 | 5

Model Comparisons

Comparison					
Model		Model	χ^2	df	p
1	-	2	5.33	3	0.149
2	-	3	27.31	15	0.026

Model Specific ResultsModel 1Model 2Model 3

Model Coefficients - AttitudeTotal

		95% Confidence Interval						95% Confidence Interval	
Predictor	Estimate	Lower	Upper	SE	Z	p	Odds ratio	Lower	Upper
SancMean	-0.0183	-0.194	0.157	0.0895	-0.204	0.838	0.982	0.824	1.171
AuthMean	-0.7398	-0.967	-0.514	0.1155	-6.404	<.001	0.477	0.380	0.598
LoyalMean	-0.1159	-0.333	0.101	0.1108	-1.046	0.295	0.891	0.716	1.106
FairMean	0.8666	0.615	1.122	0.1292	6.707	<.001	2.379	1.849	3.070
CareMean	0.5336	0.301	0.768	0.1192	4.478	<.001	1.705	1.351	2.156

[3]

Model Coefficients - AttitudeTotal

		95% Confidence Interval						95% Confidence Interval	
Predictor	Estimate	Lower	Upper	SE	Z	p	Odds ratio	Lower	Upper
SancMean	-0.0113	-0.187	0.1651	0.0898	-0.126	0.900	0.989	0.829	1.179
AuthMean	-0.7528	-0.982	-0.5261	0.1161	-6.483	<.001	0.471	0.375	0.591
LoyalMean	-0.1191	-0.337	0.0984	0.1111	-1.072	0.284	0.888	0.714	1.103
FairMean	0.8576	0.605	1.1134	0.1296	6.617	<.001	2.358	1.831	3.045
CareMean	0.5369	0.304	0.7719	0.1193	4.500	<.001	1.711	1.355	2.164
Condition:									
1 – 0	0.0421	-0.317	0.4008	0.1829	0.230	0.818	1.043	0.729	1.493
2 – 0	-0.3102	-0.668	0.0465	0.1821	-1.704	0.088	0.733	0.513	1.048
3 – 0	-0.2158	-0.580	0.1476	0.1855	-1.163	0.245	0.806	0.560	1.159

[3]

Model Coefficients - AttitudeTotal

Predictor	Estimate	95% Confidence Interval		SE	Z	p	Odds ratio	95% Confidence Interval	
		Lower	Upper					Lower	Upper
SancMean	0.0268	-0.3553	0.4105	0.195	0.1372	0.891	1.027	0.701	1.508
AuthMean	-0.9506	-1.4514	-0.4578	0.253	-3.7614	<.001	0.387	0.234	0.633
LoyalMean	0.1105	-0.3743	0.5917	0.246	0.4488	0.654	1.117	0.688	1.807
FairMean	1.1517	0.6043	1.7014	0.279	4.1264	<.001	3.164	1.830	5.482
CareMean	0.8787	0.3951	1.3739	0.249	3.5253	<.001	2.408	1.485	3.951
Condition:									
1 – 0	2.6629	0.1261	5.2128	1.296	2.0551	0.040	14.338	1.134	183.609
2 – 0	2.7619	0.4298	5.1141	1.193	2.3150	0.021	15.830	1.537	166.356
3 – 0	3.3691	0.8602	5.9065	1.286	2.6205	0.009	29.052	2.364	367.403
SancMean ┬ Condition:									
SancMean ┬ (1 – 0)	0.2173	-0.3102	0.7466	0.269	0.8069	0.420	1.243	0.733	2.110
SancMean ┬ (2 – 0)	-0.2620	-0.7718	0.2450	0.259	-1.0112	0.312	0.770	0.462	1.278
SancMean ┬ (3 – 0)	-0.0379	-0.5622	0.4854	0.267	-0.1419	0.887	0.963	0.570	1.625
AuthMean ┬ Condition:									
AuthMean ┬ (1 – 0)	-0.0331	-0.7140	0.6496	0.347	-0.0953	0.924	0.967	0.490	1.915
AuthMean ┬ (2 – 0)	0.5627	-0.0745	1.2040	0.326	1.7278	0.084	1.755	0.928	3.333
AuthMean ┬ (3 – 0)	-0.0142	-0.6903	0.6623	0.344	-0.0413	0.967	0.986	0.501	1.939
LoyalMean ┬ Condition:									
LoyalMean ┬ (1 – 0)	-0.4722	-1.1067	0.1614	0.323	-1.4606	0.144	0.624	0.331	1.175
LoyalMean ┬ (2 – 0)	-0.0962	-0.7561	0.5664	0.337	-0.2853	0.775	0.908	0.469	1.762
LoyalMean ┬ (3 – 0)	-0.2127	-0.8650	0.4419	0.333	-0.6385	0.523	0.808	0.421	1.556
FairMean ┬ Condition:									
FairMean ┬ (1 – 0)	-0.2235	-0.9660	0.5259	0.380	-0.5881	0.556	0.800	0.381	1.692
FairMean ┬ (2 – 0)	-0.4532	-1.1636	0.2611	0.363	-1.2492	0.212	0.636	0.312	1.298
FairMean ┬ (3 – 0)	-0.3626	-1.1183	0.3944	0.385	-0.9407	0.347	0.696	0.327	1.484
CareMean ┬ Condition:									
CareMean ┬ (1 – 0)	-0.2729	-0.9613	0.4099	0.349	-0.7813	0.435	0.761	0.382	1.507
CareMean ┬ (2 – 0)	-0.5571	-1.2240	0.0978	0.336	-1.6558	0.098	0.573	0.294	1.103
CareMean ┬ (3 – 0)	-0.4312	-1.1125	0.2430	0.345	-1.2488	0.212	0.650	0.329	1.275

[3]

References

[32] The jamovi project (2020). *jamovi*. (Version 1.2) [Computer Software]. Retrieved from https://www.jamovi.org.

[33] R Core Team (2019). *R: A Language and environment for statistical computing*. (Version 3.6) [Computer software]. Retrieved from https://cran.r-project.org/.

[34] Ripley, B., Venables W., Bates, D. M., Hornik, K., Gebhardt, A., & Firth, D. (2018). *MASS: Support Functions and Datasets for Venables and Ripley's MASS*. [R package]. Retrieved from https://cran.r-project.org/package=MASS.

Analysis S3

Binomial Logistic Regression (software used [32–34])

Model Fit Measures

Model	Deviance	AIC	R^2_{McF}
1	.	.	.

Model Coefficients - …

Predictor	Estimate	SE	Z	p
Intercept	.	.	.	.
Condition:				
1 – 0	.	.	.	.
2 – 0	.	.	.	.
3 – 0	.	.	.	.
4 – 0	.	.	.	.
CareMean	.	.	.	.
FairMean	.	.	.	.
LoyalMean	.	.	.	.
AuthMean	.	.	.	.
SancMean	.	.	.	.

Note. Estimates represent the log odds of …

Ordinal Logistic Regression

Model Fit Measures

Model	Deviance	AIC	R^2_{McF}
1	1925	1939	0.0319
2	1923	1945	0.0329
3	1901	1963	0.0440

Note. The dependent variable 'BehaviouralMeasurePetition' has the following order: -1 | 0 | 1

Model Comparisons

Comparison					
Model		Model	χ^2	df	p
1	-	2	1.97	4	0.741
2	-	3	22.12	20	0.334

Model Specific ResultsModel 1Model 2Model 3

Model Coefficients - BehaviouralMeasurePetition

Predictor	Estimate	95% Confidence Interval		SE	Z	p	Odds ratio	95% Confidence Interval	
		Lower	Upper					Lower	Upper
SancMean	-0.0306	-0.201	0.140	0.0871	-0.351	0.725	0.970	0.818	1.151
AuthMean	-0.3906	-0.607	-0.177	0.1096	-3.563	<.001	0.677	0.545	0.838
LoyalMean	0.0721	-0.127	0.272	0.1016	0.709	0.478	1.075	0.881	1.312
FairMean	0.4863	0.251	0.725	0.1207	4.028	<.001	1.626	1.285	2.064
CareMean	0.0519	-0.162	0.267	0.1092	0.476	0.634	1.053	0.851	1.306

[3]

Model Coefficients - BehaviouralMeasurePetition

Predictor	Estimate	95% Confidence Interval		SE	Z	p	Odds ratio	95% Confidence Interval	
		Lower	Upper					Lower	Upper
SancMean	-0.0208	-0.192	0.151	0.0875	-0.238	0.812	0.979	0.825	1.163
AuthMean	-0.4073	-0.625	-0.192	0.1105	-3.687	<.001	0.665	0.535	0.825
LoyalMean	0.0752	-0.125	0.276	0.1020	0.737	0.461	1.078	0.883	1.317
FairMean	0.4943	0.258	0.734	0.1213	4.075	<.001	1.639	1.294	2.083
CareMean	0.0434	-0.172	0.260	0.1099	0.395	0.693	1.044	0.842	1.297
Condition:									
0 – 4	0.1747	-0.208	0.558	0.1953	0.895	0.371	1.191	0.812	1.747
1 – 4	0.2203	-0.159	0.601	0.1938	1.137	0.256	1.246	0.853	1.824
2 – 4	0.1976	-0.181	0.577	0.1935	1.021	0.307	1.218	0.834	1.782
3 – 4	0.0543	-0.329	0.438	0.1954	0.278	0.781	1.056	0.720	1.549

[3]

Model Coefficients - BehaviouralMeasurePetition

		95% Confidence Interval						95% Confidence Interval	
**Predictor**	**Estimate**	**Lower**	**Upper**	**SE**	**Z**	**p**	**Odds ratio**	**Lower**	**Upper**
SancMean	0.08922	-0.2935	0.4763	0.196	0.4563	0.648	1.093	0.7456	1.61
AuthMean	-0.24407	-0.7344	0.2386	0.247	-0.9871	0.324	0.783	0.4798	1.27
LoyalMean	0.03002	-0.3690	0.4278	0.202	0.1482	0.882	1.030	0.6915	1.53
FairMean	0.42062	-0.0869	0.9477	0.263	1.6012	0.109	1.523	0.9167	2.58
CareMean	-0.16044	-0.6231	0.2929	0.232	-0.6908	0.490	0.852	0.5363	1.34
Condition:									
0 – 4	-0.49425	-3.0164	2.0179	1.281	-0.3859	0.700	0.610	0.0490	7.52
1 – 4	-0.35752	-2.7070	1.9908	1.195	-0.2992	0.765	0.699	0.0667	7.32
2 – 4	0.46527	-1.7221	2.6497	1.112	0.4184	0.676	1.592	0.1787	14.15
3 – 4	-1.75677	-4.1728	0.6310	1.222	-1.4376	0.151	0.173	0.0154	1.88
SancMean ┬ Condition:									
SancMean ┬ (0 – 4)	-0.20896	-0.7775	0.3563	0.289	-0.7241	0.469	0.811	0.4596	1.43
SancMean ┬ (1 – 4)	-0.00858	-0.5460	0.5280	0.273	-0.0314	0.975	0.991	0.5793	1.70
SancMean ┬ (2 – 4)	-0.11707	-0.6627	0.4298	0.278	-0.4211	0.674	0.890	0.5154	1.54
SancMean ┬ (3 – 4)	-0.25821	-0.8121	0.2916	0.281	-0.9192	0.358	0.772	0.4439	1.34
AuthMean ┬ Condition:									
AuthMean ┬ (0 – 4)	-0.70382	-1.4447	0.0211	0.373	-1.8871	0.059	0.495	0.2358	1.02
AuthMean ┬ (1 – 4)	-0.19340	-0.8881	0.4976	0.353	-0.5483	0.584	0.824	0.4114	1.64
AuthMean ┬ (2 – 4)	-0.19673	-0.8696	0.4743	0.342	-0.5750	0.565	0.821	0.4191	1.61
AuthMean ┬ (3 – 4)	0.21893	-0.4613	0.9012	0.347	0.6314	0.528	1.245	0.6304	2.46
LoyalMean ┬ Condition:									
LoyalMean ┬ (0 – 4)	0.47386	-0.1545	1.1119	0.322	1.4700	0.142	1.606	0.8568	3.04
LoyalMean ┬ (1 – 4)	0.23851	-0.3462	0.8285	0.299	0.7975	0.425	1.269	0.7073	2.29
LoyalMean ┬ (2 – 4)	-0.28374	-0.9193	0.3476	0.322	-0.8799	0.379	0.753	0.3988	1.42
LoyalMean ┬ (3 – 4)	-0.13946	-0.7611	0.4819	0.316	-0.4407	0.659	0.870	0.4672	1.62
FairMean ┬ Condition:									
FairMean ┬ (0 – 4)	-0.04895	-0.8359	0.7376	0.400	-0.1222	0.903	0.952	0.4335	2.09
FairMean ┬ (1 – 4)	0.08748	-0.6489	0.8215	0.374	0.2338	0.815	1.091	0.5226	2.27
FairMean ┬ (2 – 4)	0.07287	-0.6431	0.7827	0.363	0.2009	0.841	1.076	0.5256	2.19
FairMean ┬ (3 – 4)	0.41275	-0.3744	1.2050	0.402	1.0270	0.304	1.511	0.6877	3.34
CareMean ┬ Condition:									
CareMean ┬ (0 – 4)	0.56348	-0.1317	1.2715	0.357	1.5781	0.115	1.757	0.8766	3.57
CareMean ┬ (1 – 4)	0.06486	-0.6130	0.7472	0.346	0.1874	0.851	1.067	0.5417	2.11

Model Coefficients - BehaviouralMeasurePetition

		95% Confidence Interval						95% Confidence Interval	
Predictor	Estimate	Lower	Upper	SE	Z	p	Odds ratio	Lower	Upper
CareMean ┬ (2 – 4)	0.26188	-0.3796	0.9213	0.330	0.7924	0.428	1.299	0.6841	2.51
CareMean ┬ (3 – 4)	0.18172	-0.5084	0.8799	0.353	0.5146	0.607	1.199	0.6014	2.41

[3]

References

[32] The jamovi project (2020). *jamovi*. (Version 1.2) [Computer Software]. Retrieved from https://www.jamovi.org.

[33] R Core Team (2019). *R: A Language and environment for statistical computing*. (Version 3.6) [Computer software]. Retrieved from https://cran.r-project.org/.

[34] Ripley, B., Venables W., Bates, D. M., Hornik, K., Gebhardt, A., & Firth, D. (2018). *MASS: Support Functions and Datasets for Venables and Ripley's MASS*. [R package]. Retrieved from https://cran.r-project.org/package=MASS.

Analysis S4

Binomial Logistic Regression

(software used [32–34])

Model Fit Measures

Model	Deviance	AIC	R^2_{McF}
1	.	.	.

Model Coefficients - …

Predictor	Estimate	SE	Z	p
Intercept	.	.	.	.
Condition:				
1 – 0	.	.	.	.
2 – 0	.	.	.	.
3 – 0	.	.	.	.
CareMean	.	.	.	.
FairMean	.	.	.	.
LoyalMean	.	.	.	.
AuthMean	.	.	.	.
SancMean	.	.	.	.

Note. Estimates represent the log odds of …

Ordinal Logistic Regression

Model Fit Measures

Model	Deviance	AIC	R^2_{McF}
1	1503	1517	0.0404
2	1502	1522	0.0410
3	1485	1535	0.0520

Note. The dependent variable 'BehaviouralMeasurePetition' has the following order: -1 | 0 | 1

Model Comparisons

Comparison					
Model		Model	χ^2	df	p
1	-	2	0.883	3	0.830
2	-	3	17.309	15	0.301

Model Specific ResultsModel 1Model 2Model 3

Model Coefficients - BehaviouralMeasurePetition

Predictor	Estimate	95% Confidence Interval Lower	95% Confidence Interval Upper	SE	Z	p	Odds ratio	95% Confidence Interval Lower	95% Confidence Interval Upper
SancMean	-0.0588	-0.251	0.134	0.0981	-0.599	0.549	0.943	0.778	1.143
AuthMean	-0.4287	-0.672	-0.189	0.1233	-3.478	<.001	0.651	0.510	0.828
LoyalMean	0.0892	-0.142	0.321	0.1180	0.756	0.450	1.093	0.868	1.379
FairMean	0.5124	0.245	0.783	0.1370	3.740	<.001	1.669	1.278	2.188
CareMean	0.0982	-0.146	0.346	0.1254	0.783	0.434	1.103	0.864	1.413

[3]

Model Coefficients - BehaviouralMeasurePetition

Predictor	Estimate	95% Confidence Interval Lower	95% Confidence Interval Upper	SE	Z	p	Odds ratio	95% Confidence Interval Lower	95% Confidence Interval Upper
SancMean	-0.0517	-0.245	0.142	0.0985	-0.526	0.599	0.950	0.783	1.152
AuthMean	-0.4396	-0.685	-0.198	0.1240	-3.545	<.001	0.644	0.504	0.820
LoyalMean	0.0911	-0.141	0.324	0.1184	0.770	0.441	1.095	0.869	1.383
FairMean	0.5081	0.241	0.779	0.1370	3.709	<.001	1.662	1.273	2.179
CareMean	0.1029	-0.142	0.351	0.1257	0.818	0.413	1.108	0.867	1.421
Condition:									
1 – 0	0.0576	-0.331	0.447	0.1982	0.291	0.771	1.059	0.718	1.563
2 – 0	0.0337	-0.354	0.421	0.1975	0.171	0.864	1.034	0.702	1.524
3 – 0	-0.1147	-0.507	0.277	0.2000	-0.574	0.566	0.892	0.602	1.320

[3]

del Coefficients - BehaviouralMeasurePetition

		95% Confidence Interval						95% Confidence Interval	
Predictor	Estimate	Lower	Upper	SE	Z	p	Odds ratio	Lower	Upper
SancMean	-0.1188	-0.5375	0.2974	0.212	-0.5606	0.575	0.888	0.5842	1.346
AuthMean	-0.9402	-1.5071	-0.4077	0.279	-3.3679	< .001	0.391	0.2216	0.665
LoyalMean	0.5002	0.0144	1.0012	0.251	1.9954	0.046	1.649	1.0145	2.722
FairMean	0.3691	-0.2189	0.9705	0.302	1.2221	0.222	1.446	0.8034	2.639
CareMean	0.3992	-0.1263	0.9405	0.271	1.4731	0.141	1.491	0.8814	2.561
Condition:									
1 – 0	0.1367	-2.5529	2.8335	1.371	0.0997	0.921	1.146	0.0779	17.006
2 – 0	0.9534	-1.5953	3.5094	1.299	0.7341	0.463	2.595	0.2029	33.427
3 – 0	-1.2525	-3.9987	1.4801	1.394	-0.8983	0.369	0.286	0.0183	4.394
SancMean ┬ Condition:									
SancMean ┬ (1 – 0)	0.1989	-0.3601	0.7607	0.285	0.6973	0.486	1.220	0.6976	2.140
SancMean ┬ (2 – 0)	0.0912	-0.4757	0.6626	0.290	0.3150	0.753	1.096	0.6214	1.940
SancMean ┬ (3 – 0)	-0.0484	-0.6232	0.5259	0.292	-0.1656	0.869	0.953	0.5362	1.692
AuthMean ┬ Condition:									
AuthMean ┬ (1 – 0)	0.5065	-0.2252	1.2508	0.375	1.3489	0.177	1.659	0.7984	3.493
AuthMean ┬ (2 – 0)	0.5029	-0.2080	1.2290	0.366	1.3758	0.169	1.654	0.8122	3.418
AuthMean ┬ (3 – 0)	0.9155	0.1972	1.6519	0.370	2.4743	0.013	2.498	1.2180	5.217
LoyalMean ┬ Condition:									
LoyalMean ┬ (1 – 0)	-0.2338	-0.8912	0.4186	0.333	-0.7014	0.483	0.791	0.4102	1.520
LoyalMean ┬ (2 – 0)	-0.7515	-1.4539	-0.0608	0.355	-2.1192	0.034	0.472	0.2337	0.941
LoyalMean ┬ (3 – 0)	-0.6089	-1.2990	0.0725	0.349	-1.7441	0.081	0.544	0.2728	1.075
FairMean ┬ Condition:									
FairMean ┬ (1 – 0)	0.1345	-0.6568	0.9251	0.403	0.3342	0.738	1.144	0.5185	2.522
FairMean ┬ (2 – 0)	0.1204	-0.6519	0.8887	0.392	0.3072	0.759	1.128	0.5211	2.432
FairMean ┬ (3 – 0)	0.4573	-0.3816	1.3018	0.428	1.0674	0.286	1.580	0.6828	3.676
CareMean ┬ Condition:									
CareMean ┬ (1 – 0)	-0.4940	-1.2311	0.2344	0.373	-1.3243	0.185	0.610	0.2920	1.264
CareMean ┬ (2 – 0)	-0.2991	-1.0034	0.4069	0.358	-0.8345	0.404	0.741	0.3666	1.502
CareMean ┬ (3 – 0)	-0.3780	-1.1263	0.3651	0.380	-0.9961	0.319	0.685	0.3242	1.441

[3]

References

[32] The jamovi project (2020). *jamovi*. (Version 1.2) [Computer Software]. Retrieved from https://www.jamovi.org.

[33] R Core Team (2019). *R: A Language and environment for statistical computing*. (Version 3.6) [Computer software]. Retrieved from https://cran.r-project.org/.

[34] Ripley, B., Venables W., Bates, D. M., Hornik, K., Gebhardt, A., & Firth, D. (2018). *MASS: Support Functions and Datasets for Venables and Ripley's MASS*. [R package]. Retrieved from https://cran.r-project.org/package=MASS.

Analysis S5

Binomial Logistic Regression (software used [32–34])

Model Fit Measures

Model	Deviance	AIC	R^2_{McF}
1	.	.	.

Model Coefficients - …

Predictor	Estimate	SE	Z	p
Intercept	.	.	.	.
Condition:				
1 – 0	.	.	.	.
2 – 0	.	.	.	.
3 – 0	.	.	.	.
4 – 0	.	.	.	.
CareMean	.	.	.	.
FairMean	.	.	.	.
LoyalMean	.	.	.	.
AuthMean	.	.	.	.
SancMean	.	.	.	.

Note. Estimates represent the log odds of …

Ordinal Logistic Regression

Model Fit Measures

Model	Deviance	AIC	R^2_{McF}
1	1925	1939	0.0319
2	1923	1945	0.0329
3	1901	1963	0.0440

Note. The dependent variable 'BehaviouralMeasurePetition' has the following order: -1 | 0 | 1

Model Comparisons

Comparison					
Model		Model	χ^2	df	p
1	-	2	1.97	4	0.741
2	-	3	22.12	20	0.334

Model Specific ResultsModel 1Model 2Model 3

Model Coefficients - BehaviouralMeasurePetition

Predictor	Estimate	95% Confidence Interval		SE	Z	p	Odds ratio	95% Confidence Interval	
		Lower	Upper					Lower	Upper
SancMean	-0.0306	-0.201	0.140	0.0871	-0.351	0.725	0.970	0.818	1.151
AuthMean	-0.3906	-0.607	-0.177	0.1096	-3.563	<.001	0.677	0.545	0.838
LoyalMean	0.0721	-0.127	0.272	0.1016	0.709	0.478	1.075	0.881	1.312
FairMean	0.4863	0.251	0.725	0.1207	4.028	<.001	1.626	1.285	2.064
CareMean	0.0519	-0.162	0.267	0.1092	0.476	0.634	1.053	0.851	1.306

[3]

Model Coefficients - BehaviouralMeasurePetition

Predictor	Estimate	95% Confidence Interval		SE	Z	p	Odds ratio	95% Confidence Interval	
		Lower	Upper					Lower	Upper
SancMean	-0.0208	-0.192	0.151	0.0875	-0.238	0.812	0.979	0.825	1.163
AuthMean	-0.4073	-0.625	-0.192	0.1105	-3.687	<.001	0.665	0.535	0.825
LoyalMean	0.0752	-0.125	0.276	0.1020	0.737	0.461	1.078	0.883	1.317
FairMean	0.4943	0.258	0.734	0.1213	4.075	<.001	1.639	1.294	2.083
CareMean	0.0434	-0.172	0.260	0.1099	0.395	0.693	1.044	0.842	1.297
Condition:									
1 – 0	0.0456	-0.341	0.432	0.1970	0.231	0.817	1.047	0.711	1.541
2 – 0	0.0228	-0.362	0.408	0.1965	0.116	0.907	1.023	0.696	1.504
3 – 0	-0.1204	-0.511	0.269	0.1989	-0.605	0.545	0.887	0.600	1.309
4 – 0	-0.1747	-0.558	0.208	0.1953	-0.895	0.371	0.840	0.572	1.231

[3]

Model Coefficients - BehaviouralMeasurePetition

		95% Confidence Interval						95% Confidence Interval	
**Predictor**	**Estimate**	**Lower**	**Upper**	**SE**	**Z**	**p**	**Odds ratio**	**Lower**	**Upper**
SancMean	-0.1197	-0.5388	0.2970	0.212	-0.5642	0.573	0.887	0.5834	1.346
AuthMean	-0.9479	-1.5150	-0.4149	0.279	-3.3935	< .001	0.388	0.2198	0.660
LoyalMean	0.5039	0.0177	1.0051	0.251	2.0090	0.045	1.655	1.0178	2.732
FairMean	0.3717	-0.2164	0.9738	0.302	1.2299	0.219	1.450	0.8054	2.648
CareMean	0.4030	-0.1231	0.9443	0.271	1.4862	0.137	1.496	0.8841	2.571
Condition:									
1 – 0	0.1368	-2.5546	2.8359	1.372	0.0997	0.921	1.147	0.0777	17.046
2 – 0	0.9596	-1.5915	3.5181	1.300	0.7381	0.460	2.611	0.2036	33.720
3 – 0	-1.2625	-4.0107	1.4723	1.395	-0.9048	0.366	0.283	0.0181	4.359
4 – 0	0.4943	-2.0179	3.0164	1.281	0.3859	0.700	1.639	0.1329	20.418
SancMean ⊤ Condition:									
SancMean ⊤ (1 – 0)	0.2004	-0.3593	0.7627	0.286	0.7018	0.483	1.222	0.6982	2.144
SancMean ⊤ (2 – 0)	0.0919	-0.4758	0.6639	0.290	0.3169	0.751	1.096	0.6214	1.942
SancMean ⊤ (3 – 0)	-0.0492	-0.6246	0.5256	0.293	-0.1682	0.866	0.952	0.5355	1.691
SancMean ⊤ (4 – 0)	0.2090	-0.3563	0.7775	0.289	0.7241	0.469	1.232	0.7003	2.176
AuthMean ⊤ Condition:									
AuthMean ⊤ (1 – 0)	0.5104	-0.2220	1.2552	0.376	1.3583	0.174	1.666	0.8010	3.509
AuthMean ⊤ (2 – 0)	0.5071	-0.2045	1.2336	0.366	1.3863	0.166	1.661	0.8151	3.434
AuthMean ⊤ (3 – 0)	0.9228	0.2038	1.6595	0.370	2.4922	0.013	2.516	1.2261	5.257
AuthMean ⊤ (4 – 0)	0.7039	-0.0211	1.4447	0.373	1.8872	0.059	2.022	0.9791	4.241
LoyalMean ⊤ Condition:									
LoyalMean ⊤ (1 – 0)	-0.2354	-0.8932	0.4177	0.334	-0.7055	0.481	0.790	0.4093	1.519
LoyalMean ⊤ (2 – 0)	-0.7577	-1.4604	-0.0663	0.355	-2.1349	0.033	0.469	0.2321	0.936
LoyalMean ⊤ (3 – 0)	-0.6134	-1.3037	0.0687	0.349	-1.7557	0.079	0.542	0.2715	1.071
LoyalMean ⊤ (4 – 0)	-0.4739	-1.1119	0.1545	0.322	-1.4701	0.142	0.623	0.3289	1.167
FairMean ⊤ Condition:									
FairMean ⊤ (1 – 0)	0.1364	-0.6561	0.9272	0.403	0.3385	0.735	1.146	0.5189	2.527
FairMean ⊤ (2 – 0)	0.1218	-0.6517	0.8904	0.392	0.3103	0.756	1.130	0.5212	2.436
FairMean ⊤ (3 – 0)	0.4616	-0.3784	1.3066	0.429	1.0766	0.282	1.587	0.6850	3.694
FairMean ⊤ (4 – 0)	0.0489	-0.7376	0.8359	0.400	0.1221	0.903	1.050	0.4782	2.307
CareMean ⊤ Condition:									
CareMean ⊤ (1 – 0)	-0.4986	-1.2359	0.2308	0.373	-1.3356	0.182	0.607	0.2906	1.260
CareMean ⊤ (2 – 0)	-0.3016	-1.0062	0.4056	0.359	-0.8404	0.401	0.740	0.3656	1.500

Model Coefficients - BehaviouralMeasurePetition

		95% Confidence Interval						95% Confidence Interval	
Predictor	Estimate	Lower	Upper	SE	Z	p	Odds ratio	Lower	Upper
CareMean ┬ (3 – 0)	-0.3817	-1.1303	0.3624	0.380	-1.0049	0.315	0.683	0.3229	1.437
CareMean ┬ (4 – 0)	-0.5634	-1.2715	0.1317	0.357	-1.5780	0.115	0.569	0.2804	1.141

[3]

Binomial Logistic Regression

Model Fit Measures

Model	Deviance	AIC	R^2_{McF}
1	1261	1273	0.0959
2	1257	1277	0.0988
3	1235	1295	0.1147

Model Comparisons

Comparison					
Model		Model	χ^2	df	p
1	-	2	4.12	4	0.390
2	-	3	22.16	20	0.332

Model Specific ResultsModel 1Model 2Model 3

Model Coefficients - BehaviouralMeasure2Donation

						95% Confidence Interval	
Predictor	Estimate	SE	Z	p	Odds ratio	Lower	Upper
Intercept	-2.5511	0.4512	-5.654	< .001	0.0780	0.0322	0.189
CareMean	0.7148	0.1323	5.404	< .001	2.0437	1.5770	2.649
FairMean	0.2826	0.1363	2.074	0.038	1.3266	1.0157	1.733
LoyalMean	-0.1277	0.1141	-1.120	0.263	0.8801	0.7038	1.101
AuthMean	-0.4617	0.1231	-3.751	< .001	0.6302	0.4952	0.802
SancMean	0.0579	0.0971	0.596	0.551	1.0596	0.8759	1.282

Note. Estimates represent the log odds of "BehaviouralMeasure2Donation = 1" vs. "BehaviouralMeasure2Donation = 0"

Model Coefficients - BehaviouralMeasure2Donation

						95% Confidence Interval	
**Predictor**	**Estimate**	**SE**	**Z**	**p**	**Odds ratio**	**Lower**	**Upper**
Intercept	-2.6975	0.4851	-5.5608	< .001	0.0674	0.0260	0.174
CareMean	0.7451	0.1336	5.5793	< .001	2.1067	1.6215	2.737
FairMean	0.2588	0.1372	1.8864	0.059	1.2953	0.9900	1.695
LoyalMean	-0.1220	0.1142	-1.0679	0.286	0.8852	0.7076	1.107
AuthMean	-0.4570	0.1238	-3.6907	< .001	0.6332	0.4968	0.807
SancMean	0.0526	0.0977	0.5383	0.590	1.0540	0.8703	1.276
Condition:							
1 – 0	0.1792	0.2161	0.8293	0.407	1.1963	0.7832	1.827
2 – 0	-0.0147	0.2158	-0.0682	0.946	0.9854	0.6455	1.504
3 – 0	0.0181	0.2169	0.0833	0.934	1.0182	0.6656	1.558
4 – 0	0.3394	0.2137	1.5884	0.112	1.4041	0.9237	2.135

Note. Estimates represent the log odds of "BehaviouralMeasure2Donation = 1" vs. "BehaviouralMeasure2Donation = 0"

Model Coefficients - BehaviouralMeasure2Donation

Predictor	Estimate	SE	Z	p	Odds ratio	95% Confidence Interval Lower	95% Confidence Interval Upper
Intercept	-3.0546	1.142	-2.674	0.007	0.0471	0.00502	0.442
CareMean	0.5463	0.290	1.882	0.060	1.7269	0.97749	3.051
FairMean	0.3842	0.320	1.202	0.229	1.4685	0.78493	2.747
LoyalMean	-0.3324	0.265	-1.255	0.210	0.7172	0.42671	1.205
AuthMean	-0.2311	0.282	-0.820	0.412	0.7937	0.45683	1.379
SancMean	0.2834	0.219	1.296	0.195	1.3276	0.86501	2.038
Condition:							
1 – 0	1.1918	1.550	0.769	0.442	3.2930	0.15789	68.680
2 – 0	1.6270	1.467	1.109	0.267	5.0888	0.28694	90.249
3 – 0	-0.6524	1.574	-0.415	0.679	0.5208	0.02382	11.387
4 – 0	-0.2015	1.517	-0.133	0.894	0.8175	0.04177	15.997
CareMean ┬ Condition:							
CareMean ┬ (1 – 0)	0.2413	0.437	0.553	0.580	1.2730	0.54097	2.995
CareMean ┬ (2 – 0)	0.3057	0.437	0.700	0.484	1.3576	0.57698	3.194
CareMean ┬ (3 – 0)	0.1169	0.422	0.277	0.782	1.1240	0.49113	2.572
CareMean ┬ (4 – 0)	0.4722	0.415	1.138	0.255	1.6035	0.71080	3.618
FairMean ┬ Condition:							
FairMean ┬ (1 – 0)	-0.2181	0.456	-0.478	0.632	0.8040	0.32898	1.965
FairMean ┬ (2 – 0)	-0.4459	0.441	-1.010	0.313	0.6403	0.26951	1.521
FairMean ┬ (3 – 0)	0.2098	0.458	0.458	0.647	1.2334	0.50302	3.024
FairMean ┬ (4 – 0)	-0.1552	0.439	-0.353	0.724	0.8563	0.36207	2.025
LoyalMean ┬ Condition:							
LoyalMean ┬ (1 – 0)	0.7622	0.369	2.063	0.039	2.1429	1.03876	4.421
LoyalMean ┬ (2 – 0)	0.2731	0.385	0.709	0.478	1.3141	0.61747	2.796
LoyalMean ┬ (3 – 0)	0.2009	0.377	0.533	0.594	1.2225	0.58374	2.560
LoyalMean ┬ (4 – 0)	-0.2115	0.367	-0.576	0.565	0.8093	0.39385	1.663
AuthMean ┬ Condition:							
AuthMean ┬ (1 – 0)	-1.0274	0.433	-2.373	0.018	0.3579	0.15318	0.836
AuthMean ┬ (2 – 0)	-0.4720	0.387	-1.218	0.223	0.6238	0.29192	1.333
AuthMean ┬ (3 – 0)	0.1095	0.394	0.278	0.781	1.1157	0.51581	2.413
AuthMean ┬ (4 – 0)	0.2002	0.405	0.495	0.621	1.2217	0.55258	2.701
SancMean ┬ Condition:							
SancMean ┬ (1 – 0)	-0.0515	0.313	-0.164	0.870	0.9498	0.51406	1.755
SancMean ┬ (2 – 0)	-0.2228	0.312	-0.715	0.475	0.8003	0.43462	1.474
SancMean ┬ (3 – 0)	-0.5497	0.309	-1.781	0.075	0.5771	0.31516	1.057
SancMean ┬ (4 – 0)	-0.2876	0.321	-0.895	0.371	0.7500	0.39962	1.408

Note. Estimates represent the log odds of "BehaviouralMeasure2Donation = 1" vs. "BehaviouralMeasure2Donation = 0"

References

[32] The jamovi project (2020). *jamovi*. (Version 1.2) [Computer Software]. Retrieved from https://www.jamovi.org.

[33] R Core Team (2019). *R: A Language and environment for statistical computing*. (Version 3.6) [Computer software]. Retrieved from https://cran.r-project.org/.

[34] Ripley, B., Venables W., Bates, D. M., Hornik, K., Gebhardt, A., & Firth, D. (2018). *MASS: Support Functions and Datasets for Venables and Ripley's MASS*. [R package]. Retrieved from https://cran.r-project.org/package=MASS.

Analysis S6

Binomial Logistic Regression (software used [32–34])

Model Fit Measures

Model	Deviance	AIC	R^2_{McF}
1	.	.	.

Model Coefficients - …

Predictor	Estimate	SE	Z	p
Intercept	.	.	.	.
Condition:				
1 – 0	.	.	.	.
2 – 0	.	.	.	.
3 – 0	.	.	.	.
CareMean	.	.	.	.
FairMean	.	.	.	.
LoyalMean	.	.	.	.
AuthMean	.	.	.	.
SancMean	.	.	.	.

Note. Estimates represent the log odds of …

Ordinal Logistic Regression

Model Fit Measures

Model	Deviance	AIC	R^2_{McF}
1	1503	1517	0.0404
2	1502	1522	0.0410
3	1485	1535	0.0520

Note. The dependent variable 'BehaviouralMeasurePetition' has the following order: -1 | 0 | 1

Model Comparisons

Comparison					
Model		Model	χ^2	df	p
1	-	2	0.883	3	0.830
2	-	3	17.309	15	0.301

Model Specific ResultsModel 1Model 2Model 3

Model Coefficients - BehaviouralMeasurePetition

		95% Confidence Interval						95% Confidence Interval	
Predictor	Estimate	Lower	Upper	SE	Z	p	Odds ratio	Lower	Upper
SancMean	-0.0588	-0.251	0.134	0.0981	-0.599	0.549	0.943	0.778	1.143
AuthMean	-0.4287	-0.672	-0.189	0.1233	-3.478	<.001	0.651	0.510	0.828
LoyalMean	0.0892	-0.142	0.321	0.1180	0.756	0.450	1.093	0.868	1.379
FairMean	0.5124	0.245	0.783	0.1370	3.740	<.001	1.669	1.278	2.188
CareMean	0.0982	-0.146	0.346	0.1254	0.783	0.434	1.103	0.864	1.413

[3]

Model Coefficients - BehaviouralMeasurePetition

		95% Confidence Interval						95% Confidence Interval	
Predictor	Estimate	Lower	Upper	SE	Z	p	Odds ratio	Lower	Upper
SancMean	-0.0517	-0.245	0.142	0.0985	-0.526	0.599	0.950	0.783	1.152
AuthMean	-0.4396	-0.685	-0.198	0.1240	-3.545	<.001	0.644	0.504	0.820
LoyalMean	0.0911	-0.141	0.324	0.1184	0.770	0.441	1.095	0.869	1.383
FairMean	0.5081	0.241	0.779	0.1370	3.709	<.001	1.662	1.273	2.179
CareMean	0.1029	-0.142	0.351	0.1257	0.818	0.413	1.108	0.867	1.421
Condition:									
1 – 0	0.0576	-0.331	0.447	0.1982	0.291	0.771	1.059	0.718	1.563
2 – 0	0.0337	-0.354	0.421	0.1975	0.171	0.864	1.034	0.702	1.524
3 – 0	-0.1147	-0.507	0.277	0.2000	-0.574	0.566	0.892	0.602	1.320

[3]

Model Coefficients - BehaviouralMeasurePetition

		95% Confidence Interval						95% Confidence Interval	
Predictor	Estimate	Lower	Upper	SE	Z	p	Odds ratio	Lower	Upper
SancMean	-0.1188	-0.5375	0.2974	0.212	-0.5606	0.575	0.888	0.5842	1.346
AuthMean	-0.9402	-1.5071	-0.4077	0.279	-3.3679	<.001	0.391	0.2216	0.665
LoyalMean	0.5002	0.0144	1.0012	0.251	1.9954	0.046	1.649	1.0145	2.722
FairMean	0.3691	-0.2189	0.9705	0.302	1.2221	0.222	1.446	0.8034	2.639
CareMean	0.3992	-0.1263	0.9405	0.271	1.4731	0.141	1.491	0.8814	2.561
Condition:									
1 – 0	0.1367	-2.5529	2.8335	1.371	0.0997	0.921	1.146	0.0779	17.006
2 – 0	0.9534	-1.5953	3.5094	1.299	0.7341	0.463	2.595	0.2029	33.427
3 – 0	-1.2525	-3.9987	1.4801	1.394	-0.8983	0.369	0.286	0.0183	4.394
SancMean ┬ Condition:									
SancMean ┬ (1 – 0)	0.1989	-0.3601	0.7607	0.285	0.6973	0.486	1.220	0.6976	2.140
SancMean ┬ (2 – 0)	0.0912	-0.4757	0.6626	0.290	0.3150	0.753	1.096	0.6214	1.940
SancMean ┬ (3 – 0)	-0.0484	-0.6232	0.5259	0.292	-0.1656	0.869	0.953	0.5362	1.692
AuthMean ┬ Condition:									
AuthMean ┬ (1 – 0)	0.5065	-0.2252	1.2508	0.375	1.3489	0.177	1.659	0.7984	3.493
AuthMean ┬ (2 – 0)	0.5029	-0.2080	1.2290	0.366	1.3758	0.169	1.654	0.8122	3.418
AuthMean ┬ (3 – 0)	0.9155	0.1972	1.6519	0.370	2.4743	0.013	2.498	1.2180	5.217
LoyalMean ┬ Condition:									
LoyalMean ┬ (1 – 0)	-0.2338	-0.8912	0.4186	0.333	-0.7014	0.483	0.791	0.4102	1.520
LoyalMean ┬ (2 – 0)	-0.7515	-1.4539	-0.0608	0.355	-2.1192	0.034	0.472	0.2337	0.941
LoyalMean ┬ (3 – 0)	-0.6089	-1.2990	0.0725	0.349	-1.7441	0.081	0.544	0.2728	1.075
FairMean ┬ Condition:									
FairMean ┬ (1 – 0)	0.1345	-0.6568	0.9251	0.403	0.3342	0.738	1.144	0.5185	2.522
FairMean ┬ (2 – 0)	0.1204	-0.6519	0.8887	0.392	0.3072	0.759	1.128	0.5211	2.432
FairMean ┬ (3 – 0)	0.4573	-0.3816	1.3018	0.428	1.0674	0.286	1.580	0.6828	3.676
CareMean ┬ Condition:									
CareMean ┬ (1 – 0)	-0.4940	-1.2311	0.2344	0.373	-1.3243	0.185	0.610	0.2920	1.264
CareMean ┬ (2 – 0)	-0.2991	-1.0034	0.4069	0.358	-0.8345	0.404	0.741	0.3666	1.502
CareMean ┬ (3 – 0)	-0.3780	-1.1263	0.3651	0.380	-0.9961	0.319	0.685	0.3242	1.441

[3]

Binomial Logistic Regression

Model Fit Measures

Model	Deviance	AIC	R^2_{McF}
1	999	1011	0.0926
2	998	1016	0.0935
3	981	1029	0.1087

Model Comparisons

Comparison					
Model		Model	χ^2	df	p
1	-	2	1.07	3	0.784
2	-	3	16.72	15	0.336

Model Specific ResultsModel 1Model 2Model 3

Model Coefficients - BehaviouralMeasure2Donation

						95% Confidence Interval	
Predictor	Estimate	SE	Z	p	Odds ratio	Lower	Upper
Intercept	-2.3913	0.510	-4.687	< .001	0.0915	0.0337	0.249
CareMean	0.6743	0.151	4.465	< .001	1.9626	1.4598	2.639
FairMean	0.2628	0.155	1.695	0.090	1.3005	0.9598	1.762
LoyalMean	-0.0188	0.130	-0.145	0.885	0.9814	0.7606	1.266
AuthMean	-0.5540	0.138	-4.004	< .001	0.5746	0.4381	0.754
SancMean	0.0568	0.108	0.526	0.599	1.0584	0.8566	1.308

Note. Estimates represent the log odds of "BehaviouralMeasure2Donation = 1" vs. "BehaviouralMeasure2Donation = 0"

Model Coefficients - BehaviouralMeasure2Donation

						95% Confidence Interval	
Predictor	Estimate	SE	Z	p	Odds ratio	Lower	Upper
Intercept	-2.4547	0.541	-4.5352	< .001	0.0859	0.0297	0.248
CareMean	0.6817	0.151	4.5000	< .001	1.9772	1.4693	2.661
FairMean	0.2601	0.155	1.6735	0.094	1.2970	0.9565	1.759
LoyalMean	-0.0132	0.130	-0.1012	0.919	0.9869	0.7643	1.274
AuthMean	-0.5604	0.139	-4.0297	< .001	0.5710	0.4348	0.750
SancMean	0.0587	0.108	0.5418	0.588	1.0605	0.8576	1.311
Condition:							
1 – 0	0.1804	0.216	0.8367	0.403	1.1977	0.7849	1.828
2 – 0	-0.0123	0.215	-0.0570	0.955	0.9878	0.6477	1.507
3 – 0	0.0110	0.217	0.0510	0.959	1.0111	0.6613	1.546

Note. Estimates represent the log odds of "BehaviouralMeasure2Donation = 1" vs. "BehaviouralMeasure2Donation = 0"

Model Coefficients - BehaviouralMeasure2Donation

						95% Confidence Interval	
Predictor	Estimate	SE	Z	p	Odds ratio	Lower	Upper
Intercept	-3.0546	1.142	-2.674	0.007	0.0471	0.00502	0.442
CareMean	0.5463	0.290	1.882	0.060	1.7269	0.97749	3.051
FairMean	0.3842	0.320	1.202	0.229	1.4685	0.78493	2.747
LoyalMean	-0.3324	0.265	-1.255	0.210	0.7172	0.42671	1.205
AuthMean	-0.2311	0.282	-0.820	0.412	0.7937	0.45683	1.379
SancMean	0.2834	0.219	1.296	0.195	1.3276	0.86501	2.038
Condition:							
1 – 0	1.1918	1.550	0.769	0.442	3.2930	0.15789	68.680
2 – 0	1.6270	1.467	1.109	0.267	5.0888	0.28694	90.249
3 – 0	-0.6524	1.574	-0.415	0.679	0.5208	0.02382	11.387
CareMean ┬ Condition:							
CareMean ┬ (1 – 0)	0.2413	0.437	0.553	0.580	1.2730	0.54097	2.995
CareMean ┬ (2 – 0)	0.3057	0.437	0.700	0.484	1.3576	0.57698	3.194
CareMean ┬ (3 – 0)	0.1169	0.422	0.277	0.782	1.1240	0.49113	2.572
FairMean ┬ Condition:							
FairMean ┬ (1 – 0)	-0.2181	0.456	-0.478	0.632	0.8040	0.32898	1.965
FairMean ┬ (2 – 0)	-0.4459	0.441	-1.010	0.313	0.6403	0.26951	1.521
FairMean ┬ (3 – 0)	0.2098	0.458	0.458	0.647	1.2334	0.50302	3.024
LoyalMean ┬ Condition:							
LoyalMean ┬ (1 – 0)	0.7622	0.369	2.063	0.039	2.1429	1.03876	4.421
LoyalMean ┬ (2 – 0)	0.2731	0.385	0.709	0.478	1.3141	0.61747	2.796
LoyalMean ┬ (3 – 0)	0.2009	0.377	0.533	0.594	1.2225	0.58374	2.560
AuthMean ┬ Condition:							
AuthMean ┬ (1 – 0)	-1.0274	0.433	-2.373	0.018	0.3579	0.15318	0.836
AuthMean ┬ (2 – 0)	-0.4720	0.387	-1.218	0.223	0.6238	0.29192	1.333
AuthMean ┬ (3 – 0)	0.1095	0.394	0.278	0.781	1.1157	0.51581	2.413
SancMean ┬ Condition:							
SancMean ┬ (1 – 0)	-0.0515	0.313	-0.164	0.870	0.9498	0.51406	1.755
SancMean ┬ (2 – 0)	-0.2228	0.312	-0.715	0.475	0.8003	0.43462	1.474
SancMean ┬ (3 – 0)	-0.5497	0.309	-1.781	0.075	0.5771	0.31516	1.057

Note. Estimates represent the log odds of "BehaviouralMeasure2Donation = 1" vs. "BehaviouralMeasure2Donation = 0"

References

[32] The jamovi project (2020). *jamovi*. (Version 1.2) [Computer Software]. Retrieved from https://www.jamovi.org.

[33] R Core Team (2019). *R: A Language and environment for statistical computing*. (Version 3.6) [Computer software]. Retrieved from https://cran.r-project.org/.

[34] Ripley, B., Venables W., Bates, D. M., Hornik, K., Gebhardt, A., & Firth, D. (2018). *MASS: Support Functions and Datasets for Venables and Ripley's MASS*. [R package]. Retrieved from https://cran.r-project.org/package=MASS.
